# Prevalence and severity of physical complaints and their association with well-being among older adults in Tehran

**DOI:** 10.1186/s12877-025-06875-9

**Published:** 2025-12-16

**Authors:** Arya Hamedanchi, Fereshteh Rezaie, Asma Pourhoseingholi, Mohammadreza Lakpour, Aynaz Goodarzi, Ayoub Nafei, Hamid Hamzezadeh

**Affiliations:** 1 Shahid Beheshti University of Medical Sciences branch, ACECR, Tehran, Iran; 2 Iranian Research Center on Aging, University of Social Welfare and Rehabilitation Sciences (USWR), Tehran, Iran; 3 School of Nursing, Shahid Beheshti University of Medical Sciences, Tehran, Iran; 4 Department of Medical Education, School of Medical Education & Learning Technologies, Shahid Beheshti, University of Medical Sciences, Tehran, Iran

**Keywords:** Aging, Elderly population, Complaints, Well-being, Geriatric health

## Abstract

**Introduction:**

As people age, they experience not only physiological changes but also an increase in physical complaints. Although many of these complaints do not represent specific diseases, they cause significant suffering for older adults. This cross-sectional study aims to investigate the prevalence and severity of common physical complaints in older adults.

**Methods:**

In this cross-sectional study conducted from June to December 2024, 606 older adults aged 60 and above were selected through stratified sampling to complete a questionnaire. The questionnaire collected information on demographic characteristics, primary physical complaints, and included the WHO-5 Well-being Index. Data were analyzed using SPSS version 21.

**Results:**

The mean age of the participants was 69.6 ± 7, and 321 (53%) were female. The most common complaints were limb pain (90%), back pain (84%), weakness (81%), and fatigue (81%). Individuals aged 70 and older reported significantly higher rates of visual issues (*p* < 0.001), hearing problems (*p* < 0.001), incontinence (*p* < 0.05), and increased daytime urinary frequency (*p* < 0.05). However, distress due to hair loss or graying, and distress due to skin wrinkles were higher in the 60–69 year age group (*P* < 0.05). A negative correlation was found between the number of self-reported complaints and well-being (*r* = -0.39, *p* < 0.001).

**Conclusion:**

The result of the current study underscores the importance of addressing physical complaints in older adults. Physical complaints should be evaluated during routine assessments of older patients. There should be greater emphasis on screening, evaluating, improving, and alleviating these complaints.

## Introduction

The physiological changes and functional decline in various organs during aging increase individuals’ susceptibility to multiple disorders and diseases [1]. Although these changes are not considered diseases, they can threaten an individual’s ability to adapt [2]. In many cases, this decline is tangible, and the experience is unpleasant, often expressed through various physical complaints. These complaints should be taken seriously, as they may not reflect of normal aging but rather a specific disease that requires intervention [3]. In some cases, addressing these complaints does not yield a particular diagnosis, and only a few are related to serious diseases [4].

During the first visit, physicians typically ask patients to describe their primary concerns or chief complaints. The patient’s chief complaint is a critical diagnostic indicator. Therefore, paying attention to these chief complaints is crucial, as emphasized in medical practice guidelines [5]. A deep chest pain radiating to the back or shoulders may facilitate early diagnosis and treatment of ischemic heart disease [6]. Therefore, individuals with diabetes or opioid addiction who experience silent myocardial infarction are more likely to remain undiagnosed [7].

Complaints are considered only when they are manifestations of a specific disorder. Consequently, symptoms attributed to normal aging may remain unexplained, underestimated, or overlooked, often labeled as non-diagnostic or non-significant [8]. Meanwhile, the manifestation of certain diseases may differ in older adults, and even general complaints can be indicative of specific diseases. For example, physical complaints are common in depression. These complaints can be especially intolerable for older adults, significantly affecting their quality of life [9, 10].

They can also reflect unpleasant feelings or sensations and even affect the well-being of aged people [11]. Evidence shows that long-term chronic pain can affect well-being in older adults. These pains are also associated with a decline in physical function [12]. Back and knee pain, in particular, can significantly impact balance in older adults [13].

Sensory decline is another characteristic of normal aging that can lead to numerous difficulties in older age [14]. A study by Xiang et al. found that older adults with grater visual impairment had lower perceived well-being [15]. In addition to limitations in daily life functions, these impediments can lead to social and self-stigma, which can affect self-esteem and social interactions [16].

Urinary and gastrointestinal complaints are also common among older adults. These problems can impact their psychosocial well-being. For example, the results of Vasiee et al.‘s study spotlighted a significant association between urinary incontinence and self-esteem in older adults [17].

Gonzalez et al. argued that physical illnesses and psychological disorders, such as anxiety, depression, and psychosis, can have a bidirectional relationship. They stated that the perceived and actual severity of physical illnesses, especially gastrointestinal disorders, can exacerbate psychiatric conditions and vice versa [18].

The presence of an impairment in a system function does not necessarily when they cause severe dysfunction, they can be ignored, denied, and unexpressed by older adults [19].

**Fig. 1:**
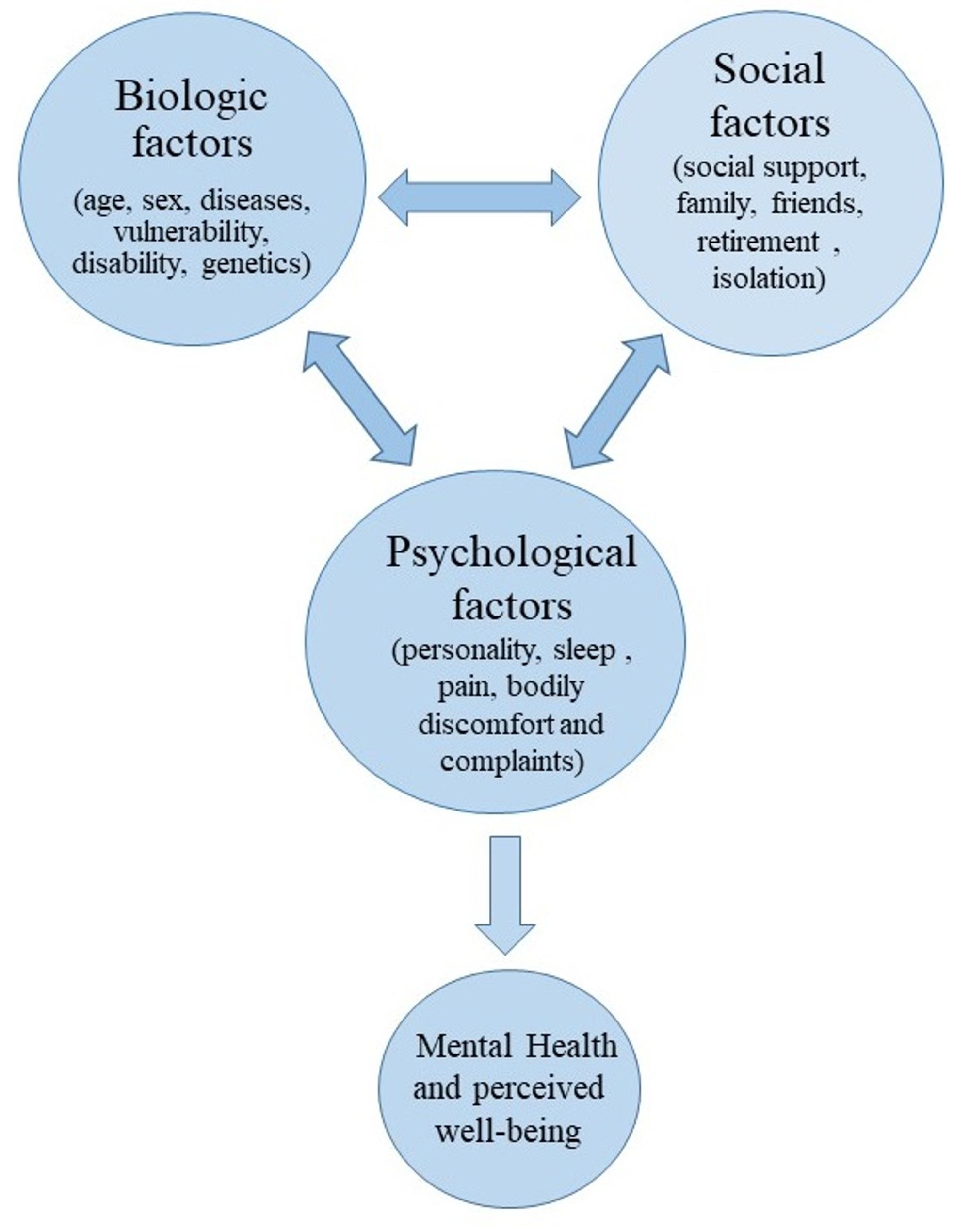
A conceptual framework for major factors influencing well-being in older adults

Well-being is a broad, multifaceted subjective concept which is defined by Tov as “all the ways in which people experience and evaluate their lives positively” [20]. Based on George Engel’s (1978) health model an holistic view of health and well-being includes physical, psychological, and social dimensions [21]. Aging is accompanied by biopsychosocial changes that can substantially influence older adults’ well-being [22]. Older people show a decline in their organ function and are more susceptible to chronic diseases. Additionally, cognition impairments and depression are common among them. Social aging can affect their participation in society leading to isolation and mental health issues [23]. Advancing well-being which has a close relation with mental health, can create active, resilient and sustainable communities at local, national and global levels [24, 25]. Figure 1 shows a conceptual framework for major factors influencing well-being in older adults.

The global increase in the number of people aged 70 and over should be highlighted. It is predicted that by 2030, individuals aged 70 and over, who will make up 8% of the world’s population, will account for more than 50% of cancer cases, 58% of diabetes cases, 95% of Alzheimer’s and dementia cases, 62% of non-infectious mortalities, and 30% of infectious diseases [26]. Consistently, life expectancy has increased over the last decades in Iran. It has risen from 44 years in 1960 to 70 years in 2000 and 76 years in 2019 [27]. Therefore, it is imperative to consider the health needs of the emerging population of people aged 70 and older in Iran.

Numerous studies have examined the primary concerns of older adults and their impact on their daily lives. However, most have focused on only one or a set of complaints, emphasizing their diagnostic value and investigating them from a medical perspective. Moreover, their data were generally collected from patients attending health centers, and the general population of older adults has not been considered. Generally, it can be claimed that these complaints are investigated objectively from a medical perspective rather than through the patients’ experiences. There are only a few available studies that examine the prevalence of common, annoying complaints among older Iranian adults. This study aimed to investigate the prevalence and severity of chief complaints among older adults in Iran and analyze their association with overall well-being.

## Methods

### Study design

This was a descriptive-analytical cross-sectional study.

### Study setting

This study was conducted in Tehran between June and December 2024. Data were collected through questionnaires completed by older adults in 22 districts of Tehran. Participants were primarily recruited from public settings, including parks and cultural centers, across Tehran’s 22 districts. Following a clear explanation of the study’s aims and procedures, informed consent was obtained from those who agreed to participate. In cases of illiteracy or visual difficulties, a questioner assisted the participant by reading the questions and recording their answers. This recruitment approach supported a reasonable level of diversity within the stratified sampling framework.

### Participants

The inclusion criteria for recruiting participants were age 60 years or older, living in Tehran, willingness to participate, and mental and physical ability to participate. The exclusion criteria included a history of acute illness or hospitalization within the last two months, being institutionalized, unwillingness to participate, or choosing to opt out. Informed consent was obtained from all participants prior to their involvement in the study.

### Sample size

The sample size was calculated using Cochran’s sample size formula [28]. Due to the absence of prior studies on the prevalence of common complaints among older adults, “q” was assumed to be 0.5. Considering a power of 0.8 and a type I error (alpha) of 0.05, along with an expected error of 0.04, the minimum sample size was determined to be 601. Stratified sampling [29] was conducted across the 22 districts of Tehran, based on the older adult population in each district. A total of 606 older adults participated in the study. District 2, with an elderly population of 125,843, had the highest number of participants (*N* = 58), while District 9, with an elderly population of 18,971, had the fewest (*N* = 10).

### Measures and variables

A questionnaire was used to collect demographic and personal information, including variables such as age, sex, education, employment status, number of dependent family members, number of people living with them, chronic diseases, and care responsibilities. The World Health Organization’s Five Well-Being Index (WHO-5) is a self-report tool used to measure mental well-being. This questionnaire contains five questions, rated on a 6-point scale (0 to 5). Higher scores indicate better mental well-being [30]. The psychometric properties of the Persian version of the WHO-5 were examined by Dehshiri and Mousavi, revealing a Cronbach’s alpha of 0.89 and a test-retest coefficient of 0.82 [31]. A checklist including 24 questions was used to evaluate the presence and severity of common physical complaints in older adults. The checklist questions were selected from the medical records of Shahid Beheshti University of Medical Sciences [32], Hazzard’s Geriatric Medicine and Gerontology [33], and Comprehensive Geriatric Assessment [34]. The contents of the complaint questions, which were rated on a 6-point scale, were confirmed by an expert panel including a geriatrician, a gerontologist, a geriatric nurse, a public health specialist, and a preventive medicine specialist. More specifically, each question regarding physical complaints included six response options scored from 0 to 5, where 0 indicates ‘not at all’, 1 ‘very mild’, 2 ‘mild’, 3 ‘moderate’, 4 ‘severe’, and 5 ‘very severe’. Higher scores reflect greater intensity of the physical complaint. The maximum possible total score across the 22 items was 120. Sex, education, retirement, chronic diseases, and insurance were considered potential confounders and were included in Model 3 of the adjusted linear regression.

### Analysis

In addition to descriptive analysis to report the mean well-being and the prevalence of each complaint among the sample population, the chi-square test was used to compare the presence of each complaint between men and women. Given the non-normality of the total scores of the complaints, the Spearman correlation coefficient was used to investigate the correlation between the complaints and the well-being score. Subsequently, a linear regression model was used to test the relation between complaints and well-being scores. Model 1 included complaint and well-being scores. Adjusted Model 2 included sex and age, while adjusted Model 3 included sex, education, retirement status, chronic diseases, and insurance. Other variables with a p-value greater than 0.2 for well-being scores were excluded from the models.

Missing values were replaced with the mean of nearby points. Finally, Cronbach’s alpha was calculated for both questionnaires- the well-being and complaint checklist- with a threshold of 0.7 or higher deemed acceptable [35].

## Results

A total of 606 individuals participated in the current cross-sectional study. Their mean age was 69.6 ± 7 and 321(53%) were female. Table 1 shows the descriptive characteristics for the studied population by gender.

**Table 1:** Demographic and baseline characteristics of the studied population

Variables	Total population	Men	women
N (%)	606	285(47)	321(53)
Age (mean ± SD)	69.6 ± 7.0	70.5 ± 7.2	68.8 ± 6.8
Education, n (%)			
Illiterate	83(13.8)	18(6.4)	65(20.3)
Primary	178(29.6)	64(22.7)	114(35.6)
High school	89(14.8)	46(16.3)	43(13.5)
College	197(32.7)	117(41.5)	80(25)
University	55(9.1)	37(13.1)	18(5.6)
Job, n (%)			
Full time	17(2.8)	14(4.9)	3(0.9)
Part time	51(8.4)	47(16.5)	4(1.3)
None	538(88.8)	224(78.6)	314(97.8)
Retired, n (%)			
Yes	315 (52%)	273 (95.8%)	42 (13.1%)
No	291 (48%)	12 (4.2%)	279 (86.9%)
Retirement age (mean ± SD)	52.3 ± 4.9	52.6 ± 4.9	49.9 ± 4.1
Dependent family members, n (%)			
none	55(9.1)	8(2.80)	47(14.6)
1	251(41.4)	122(42.8)	129(40.2)
2	171(28.2)	85(29.8)	86(26.8)
3	98(16.2)	53(18.6)	45(14.0)
4	16(2.6)	8(2.8)	8(2.5)
More than 5	15(2.5)	9(3.2)	6(1.9)
Living alone, n (%)			
Yes	60(9.9)	8(2.8)	52(16.2)
No	546(90.1)	277(97.2)	269(83.8)
Living with spouse, n (%)			
Yes	458(75.5)	259(90.8)	199(62)
No	148(24.5)	26(9.2)	122(38)
Living with one child, n (%)			
Yes	188(31)	90(31.5)	98(30.5)
No	418((69)	195(68.5)	223(69.5)
Living with two children, n (%)			
Yes	97(16)	50(17.5)	47(14.6)
No	509(84)	235(82.5)	274(85.4)
Living with three or more children, n (%)			
Yes	24(4)	12(4.2)	12(3.7)
No	582(96)	273(95.8)	309(96.3)
Living with relatives, n (%)			
Yes	35(5.7)	12(4.2)	23(7)
No	571(94.3)	273(95.8)	298(93)
Chronic disease, n (%)			
Yes	557(92)	254(89)	303(94)
No	49(8)	31(11)	18(6)
Cardiac diseases, n (%)			
Yes	174(28.7)	86(30)	88(27.4)
No	432(71.3)	199(70)	233(72.6)
Hypertension, n (%)			
Yes	277(45.7)	120(42)	157(49)
No	329(54.3)	165(58)	164(51)
Diabetes, n (%)			
Yes	219(36)	106(37)	113(35)
No	387(64)	179(63)	208(65)
Arthritis, n (%)			
Yes	234(38.6)	70(24.5)	164(51)
No	372(61.4)	215(75.5)	157(49)
Taking care of others, n (%)			
Yes	26(4.3)	11(4)	15(4.6)
No	580(95.7)	274(96)	306(95.4)

The checklist consisted of 24 questions regarding common complaints associated with aging. The initial analysis of the responses involved categorizing them into two conditions: “yes” and “no.” After that, the severity of each complaint was assessed based on the reported responses, using scoring system ranging from 0 (no complaint) to 5 (severe). The maximum total score possible was 120.

The results showed that the most common complaints were pain in the limbs (90%), back pain (84%), and weakness and fatigue (both 81%). Table 2 shows the prevalence of the complaints by gender. There was no significant difference between women and men in experiencing dry mouth, vertigo, headache, dizziness, visual problems, hearing problems, chest pain, breathlessness, itching skin, constipation, waking up at night for urination, and urinary frequency during the day (p-value > 0.05). Other complaints were significantly more common in women (p-values < 0.05) (Table 2).

**Table 2:** Frequencies of complaints by gender

Complaints	Total population *n* (%)	men *n* (%)	women *n* (%)	*p*-value
Dry mouth				
Yes	427(70)	196(69)	231(72)	0.39
No	179(30)	89(31)	90(28)	
Headache				
Yes	356(59)	159(56)	197(62)	0.15
No	249(41)	126(44)	123(38)	
Dizziness				
Yes	539(60)	162(57)	197(61)	0.26
No	247(40)	123(43)	124(39)	
Weakness				
Yes	492(81)	216(76)	276(86)	< 0.01*
No	114(19)	69(24)	45(14)	
Fatigue				
Yes	488(81)	213(75)	275(86)	< 0.01*
No	118(19)	72(25)	46(14)	
Visual problems				
Yes	460(76)	209(73)	251(78)	0.16
No	146(24)	76(27)	70(22)	
Hearing problems				
Yes	420(69)	199(70)	221(69)	0.75
No	185(31)	85(30)	100(31)	
Back pain				
Yes	510(84)	222(78)	288(90)	< 0.01*
No	96(16)	63(22)	33(10)	
Pain in the limbs				
Yes	543(90)	240(84)	303(94)	< 0.01*
No	63(10)	45(16)	18(6)	
Body pains				
Yes	425(70)	184(65)	241(75)	< 0.01*
No	180(30)	100(35)	80(25)	
Poor appetite				
Yes	266(44)	103(36)	163(51)	< 0.01*
No	340(56)	182(64)	158(49)	
Burning or heaviness in the stomach				
Yes	274(45)	104(36)	170(53)	< 0.01*
No	330(55)	181(64)	149(47)	
Chest pain				
Yes	223(37)	101(35)	122(38)	0.51
No	383(63)	184(65)	199(62)	
Breathlessness				
Yes	243(40)	116(41)	127(40)	0.78
No	363(60)	169(59)	194(60)	
Itching skin				
Yes	194(32)	88(31)	106(33)	0.59
No	411(68)	196(69)	215(67)	
Constipation				
Yes	392(65)	174(61)	218(68)	0.07
No	213(35)	111(39)	102(32)	
Insufficient sleep				
Yes	437(72)	187(66)	250(78)	< 0.01*
No	168(28)	98(34)	70(22)	
Drowsiness				
Yes	378(62)	163(57)	215(67)	0.01*
No	227(38)	122(43)	105(33)	
Numbness or tingling in the hands and feet				
Yes	389(64)	162(57)	227(71)	< 0.01*
No	216(36)	122(43)	94(29)	
Waking up at night to urinate				
Yes	460(76)	223(78)	237(74)	0.21
No	146(24)	62(22)	84(26)	
Involuntary urine loss				
Yes	167(28)	84(29)	83(26)	0.32
No	439(72)	201(76)	238(74)	
Urinary frequency during the day				
Yes	288(48)	136(48)	152(47)	0.93
No	318(52)	149(52)	169(53)	
Distress because of hair loss or graying				
Yes	204(34)	54(19)	150(47)	< 0.01*
No	402(66)	231(81)	171(53)	
Distress because of skin wrinkles				
Yes	172(28)	38(13)	134(42)	< 0.01*
No	434(72)	247(87)	187(58)	

* *p* < 0.05

Table 3 presents the severity of complaints in the total sample and among women and men. Pain in the limbs was the most severe complaint in both groups (2.74 ± 1.53). It was the most severe reported complaint in women (3.24 ± 1.36) and men (2.18 ± 1.52). The severity of complaints, namely weakness, fatigue, back pain, pain in the limbs, body pains, poor appetite, burning or heaviness in the stomach, insufficient sleep, drowsiness, numbness or tingling, in the hands and feet, distress because of hair loss or graying, distress because of skin wrinkles was significantly higher in women (p-value < 0.05).

**Table 3:** The severity of complaints by gender

	Total = 606				Women = 321				Men = 285				
	median	iqr	mean	sd	median	iqr	mean	sd	median	iqr	mean	sd	*p*-value
Dry mouth	1	3	1.5	1.35	1	3	1.48	1.31	1	3	1.53	1.39	0.84
Headache	1	2	1	1.11	1	2	1.06	1.13	1	1	0.94	1.08	0.14
Dizziness	1	2	1.06	1.16	1	2	1.11	1.15	1	2	1	1.16	0.18
Weakness	2	3	2.19	1.52	3	3	2.48	1.5	2	2	1.87	1.48	< 0.01
Fatigue	2	2	2.07	1.47	3	3	2.35	1.46	2	3	1.76	1.42	< 0.01
Visual problems	1	1	1.21	1.05	1	0	1.19	0.98	1	2	1.23	1.12	0.95
Hearing problems	1	2	1.21	1.18	1	1	1.09	1.08	1	2	1.35	1.27	0.21
Back pain	2	3	2.18	1.57	3	3	2.63	1.57	1	2	1.67	1.4	< 0.01
Pain in the limbs	3	3	2.74	1.53	4	1	3.24	1.36	2	3	2.18	1.52	< 0.01
Body pains	1	2	1.41	1.39	1	2	1.61	1.47	1	2	1.17	1.25	< 0.01
Poor appetite	0	1	0.81	1.17	1	1	0.95	1.24	0	1	0.64	1.07	< 0.01
Burning or heaviness in the stomach	0	2	1.01	1.4	1	3	1.28	1.51	0	1	0.72	1.21	< 0.01
Chest pain	0	1	0.52	0.82	0	1	0.53	0.81	0	1	0.51	0.82	0.58
Breathlessness	0	1	0.73	1.14	0	1	0.68	1.08	0	1	0.79	1.21	0.53
Itching skin	0	1	0.56	1.03	0	1	0.61	1.08	0	1	0.51	0.97	0.47
Constipation	1	3	1.59	1.49	1	3	1.67	1.48	1	3	1.51	1.50	0.14
Insufficient sleep	2	4	1.97	1.61	3	3	2.25	1.61	1	3	1.67	1.56	< 0.01
Drowsiness	1	2	1.17	1.26	1	2	1.24	1.25	1	2	1.09	1.27	0.05
Numbness or tingling in the hands and feet	1	3	1.60	1.63	2	3	1.93	1.69	1	2	1.24	1.47	< 0.01
Waking up at night to urinate	1	1	1.49	1.22	1	2	1.44	1.22	1	1	1.54	1.23	0.3
Involuntary urine loss	0	1	0.50	1.01	0	1	0.42	0.90	0	1	0.59	1.11	0.16
Urinary frequency during the day	0	2	0.92	1.21	0	1	0.89	1.18	0	2	0.95	1.24	0.72
Distress because of hair loss or graying	0	1	0.61	1.03	0	2	0.90	1.20	0	0	0.27	0.66	< 0.01
Distress because of skin wrinkles	0	1	0.54	1.04	0	1	0.84	1.22	0	0	0.20	0.64	< 0.01

Based on the Likert scale, the mean total score for complaints was 30.70 ± 13.8 for the entire sample population, 34.02 ± 13.07 for women, and 26.97 ± 13 for men. The Mann-Whitney U test revealed that this difference was statistically significant (*p*-value < 0.001).

A comparison between the 60- to 69-year-old and 70-year-old and older groups revealed significant differences in vision problems, hearing problems, involuntary urine loss, daytime urinary frequency, distress due to hair loss or graying, and distress due to skin wrinkles. Distress about hair loss or graying was higher in the 60–69 age group, while other issues, such as skin wrinkles, were more severe in those aged 70 and older (*p*-values < 0.05) (Table 4).

**Table 4:** Severity of complaints by age group

	(*N* = 606)				60–69 ys = 315				70 ys and above = 291				
	Median	iqr	Mean	sd	median	iqr	mean	sd	median	iqr	mean	sd	*p*-value
Dry mouth	1	3	1.5	1.35	1	2	1.4	1.33	1	3	1.61	1.36	0.05
Headache	1	2	1.00	1.11	1	1	1.00	1.1	1	2	1.01	1.12	0.99
Dizziness	1	2	1.06	1.16	1	2	0.99	1.06	1	2	1.13	1.25	0.39
Weakness	2	3	2.19	1.52	2	3	2.16	1.55	2	3	2.23	1.48	0.6
Fatigue	2	2	2.07	1.47	2	2	2.08	1.5	2.000	2	2.06	1.45	0.87
Vision problems	1	1	1.2	1.05	1	1	1.05	0.97	1	1	1.38	1.01	< 0.01
Hearing problems	1	2	1.21	1.18	1	1	0.78	0.86	1	2	1.68	1.3	< 0.01
Back pain	2	3	2.18	1.57	2	3	2.22	1.59	2	3	2.13	1.54	0.51
Pain in the limbs	3	3	2.74	1.53	3	3	2.64	1.58	3	3	2.84	1.47	0.14
Body aches	1	2	1.41	1.39	1	2	1.39	1.38	1	2	1.42	1.4	0.89
Poor appetite	0	1	0.81	1.17	0	1	0.74	1.13	0	1	0.87	1.21	0.23
Burning or heaviness in the stomach	0	2	1.01	1.40	0	2	1.03	1.44	0	1	1.00	1.37	0.89
Chest pain	0	1	0.52	0.82	0	1	0.46	0.73	0	1	0.59	0.9	0.13
Breathlessness	0	1	0.73	1.14	0	1	0.67	1.10	0	1	0.81	1.19	0.08
Itching skin	0	1	0.56	1.03	0	1	0.63	1.12	0	1	0.49	0.93	0.39
Constipation	1	3	1.59	1.49	1	3	1.53	1.49	1	3	1.67	1.49	0.26
Insufficient sleep	2	4	1.97	1.61	2	4	1.94	1.64	2	4	2.01	1.58	0.59
Drowsiness	1	2	1.17	1.26	1	2	1.08	1.20	1	2	1.27	1.32	0.1
Numbness or tingling in the hands and feet	1	3	1.60	1.63	1	3	1.6	1.65	1	3	1.62	1.60	0.61
Waking up at night to urinate	1	1	1.49	1.22	1	1	1.46	1.18	1	2	1.51	1.26	0.71
Involuntary urine loss	0	1	0.50	1.01	0	0	0.4	0.90	0	1	0.61	1.11	0.03
Urinary frequency during the day	0	2	0.92	1.21	0	1	0.82	1.19	1	2	1.02	1.22	0.02
Distress because of hair loss or graying	0	1	0.61	1.03	0	1	0.72	1.12	0	1	0.48	0.91	0.02
Distress because of skin wrinkles	0	1	0.54	1.04	0	1	0.65	1.11	0	0	0.43	0.95	< 0.01

The mean calculated WHO-wellbeing score was 13.16 ± 5.2 for the sample population, 12.80 ± 5.3 for women, and 13.57 ± 5 for men.

Given the non-normality of the complaint scores, Spearman correlation coefficient was used to assess the correlation between the results of the two questionnaires. The correlation coefficient was significant and negative (*r* = -0.39, p-value < 0.001). This suggests that as the complaint score increases, individuals’ well-being decreases.

In the next step, a linear regression model was used to test the effect of the complaint score on well-being, while controlling for the potential confounders. Model 1 included complaint and well-being scores. Adjusted model 2 included sex and age, while adjusted model 3 included sex, gender, education, retirement status, chronic diseases, and insurance.

Other variables with p-values greater than 0.2 for well-being scores were excluded from the models. This threshold was selected to retain potential confounders with suggestive univariate associations (*p* < 0.2), a common practice in multivariate modeling to reduce bias from omitted variables while maintaining model stability [36]. Sensitivity analyses using forward stepwise selection confirmed the robustness of the primary association. The results of three models indicated one-point increase in the total complaint score was associated with a 0.14-point decrease in the WHO-5 well-being score (Coefficient: -0.14, 95% CI: -0.18 to -0.10), indicating a moderate negative effect on well-being(p-value < 0.001) (Table 5).

**Table 5:** Ordinary linear regression analysis of the association of five well-being indices and the complaint score

Variable	Model-1 Coefficient (CI: 95%)	Model-2 Coefficient (CI: 95%)	Model-3 Coefficient (CI: 95%)
Complain score	-0.14 (-0.17,-0.11)	-0.14 (-0.17,-0.11)	-0.14 (-0.18,-0.10)

Model-1: Crude

Model-2: Adjusted for age and sex

Model-3: Adjusted for other variables, including age, sex, education, retirement, chronic disease, and insurance (as other variables had a p-value less than 0.2 in the crude model, they were not used in the adjusted model)

Finally, the Cronbach׳s alpha was calculated for both questionnaires: 0.82 for the complaints checklist and 0.93 for the well-being questionnaire.

## Discussion

This cross-sectional study examined the prevalence of common health complaints among older adults in Tehran. The findings revealed that the most frequently reported issues among participants included limb pain, back pain, weakness, and fatigue.

Moreover, several complaints appeared to be more common and severe among women, such as weakness, fatigue, back pain, limb pain, body aches, poor appetite, sensations of burning or fullness in the stomach, insufficient sleep, drowsiness, numbness or tingling in the hands and feet, and distress over hair loss or graying. Women also reported greater distress due to skin wrinkles. Visual problems, hearing problems, involuntary urine loss, and urinary frequency during the day were significantly higher in the age group of 70 and older. In contrast, distress due to hair loss or graying, as well as distress due to skin wrinkles, was lower in this age group. The results also showed a negative correlation between the complaints and well-being.

According to gerotranscedence theory, as individuals grow older, their perspective shifts from a rational and materialistic orientation to a more transcendental and cosmic outlook. This can explain why older adults experience less distress in response to their age-related physical changes such as wrinkles and graying hair [37]. More bodily pain in older women can be contributed to musculoskeletal diseases such as arthrosis which has been reported more common in this sex group. Due to an increase in the life span, the burden of chronic pain impacts women more than men [38]. On the other hand, evidence shows that older women are more likely to show psychosomatic symptoms compared to compared to men in the same situation [39]. However, the form and intensity of physical symptoms can be different based on cultural environments and factors that shape how individuals interpret bodily sensations, conceptualize illness, and engage with healthcare systems [40]. The results of a study by Firoozabadi et al. in Iran show that physical complaints, such as headache, unexplained pain and fatigue are much more common than psychological complaints among women with depression. This suggests that somatization may be more socially and culturally acceptable than directly expressing negative emotions and psychological distress in this population [41].

Chronic pain is a common and problematic complaint among older adults. It causes high levels of suffering, social isolation, and disability, resulting in greater costs and burdens on health care systems. Medical treatment of chronic pain in older adults is not usually recommended, and it is often limited because of the side effects. Chronic pain is a predictor of mortality and cognitive decline [42]. On the other hand, fatigue is another prevalent symptom, experienced in older age, which is associated with comorbidities, depression, and anxiety. It can exacerbate a sense of purposelessness in life and poor self-care [43]. A systematic review by Pana et al. demonstrated that self-reported fatigue was associated with the incidence of falls or risk of falling among seniors [44]. Another common complaint reported by older adults is sleep impairment. There is evidence indicating that sleep disorders is significantly associated with depression, anxiety, hypertension, myocardial infarction, diabetes, and cognitive decline [45].

Sha et al. studied 3,498 individuals aged 60 years and older in the United States between July 1999 and August 2001, focusing on physical complaints. Musculoskeletal pain (65%), fatigue (55%), back pain (45%), shortness of breath (41%), and difficulty sleeping (38%) were the most frequently reported physical complaints. The findings also showed that these physical symptoms predicted hospitalization and mortality within one year [46]. Patel et al. studied the co-occurrence of common symptoms in 7,609 community-dwelling adults aged 65 years and older in the United States between 2011 and 2017. They found out that pain, fatigue, breathing difficulty, sleeping difficulty, and anxiety were the most common reported symptoms (ranging from 12.6% for anxiety to 52.9% for pain). Three-fourths (75.0%) of community-dwelling older adults reported at least one of the six symptoms. Women were more likely to have more symptoms than men. Pain and fatigue were the most prevalent co-occurring symptoms (31.7%), and the most common triad of symptoms included pain, fatigue, and sleep difficulty (13.4%) [47]. In the current study, pain (limb and back) and fatigue ranked first and second respectively among older adults, and were more common in women.

In a study by Rousari et al. in Tehran among 1,251 older adults, 79.4% of the participants reported experiencing pain (87.8% of men and 71% of women). The prevalence of sleep problems was 32.7%, with a higher rate in women aged 80 and older (40.3%) [48]. Accordingly, musculoskeletal problems and sleep disorders were reported as the most common complaints among 710 older adults who participated in the study by Faryabi et al. (57.5% and 39.9%, respectively) [49]. In this regard, Mohtasham et al. studied the common complaints in 7499 older adults admitted to hospitals in Rasht, Iran. They reported that chest pain (13.9%), dyspnea (11.2%), and abdominal pain (10.4%) were the most common complaints of the admitted individuals [50]. Dyspnea (21%), chest pain (21%), and abdominal pain (6%) were also common complaints among older adults visiting the emergency departments of Sabzevar hospitals [51].

There is evidence suggesting that health-related complaints impact the well-being and quality of life of older adults. A study by Ritchie et al. involving 5,589 older adults over seven-years indicates that participants with persistent pain were more likely to experience declines in well-being [12]. Similarly, fatigue was closely related to both physical and mental health measures [52]. Well-being is primarily dependent on the psychological aspects of fatigue. In other words, fatigue can result in decreased well-being [53].

On the other hand, sleep is a predictor of subjective well-being [54]. Disrupted or poor sleep is a common complaint among older adults and can negatively impact their quality of life and daily functioning [55]. A systematic review and meta-analysis by Sella et al. also showed that self-reported sleep quality correlated with QoL, with a moderate effect size [56]. Therefore, appropriate interventions to promote health and improve sleep quality in older individuals are necessary [57]. Non-pharmaceutical treatments are preferable for older adults due to the higher side effects of sleeping pills [58].

The current study had some limitations. It mainly focused on the physical aspects of complaints. There are a variety of psychological complaints among older adults that are closely associated with physical health. Since the data were collected through self-administered questionnaires, the findings may be subject to recall and reporting biases, which could affect the accuracy of the results. Given Tehran’s distinct cultural, climatic, and socioeconomic characteristics, the findings of this study may not be fully generalizable to other regions of Iran.

## Conclusion

The result of the current study underscores the importance of addressing physical complaints in older adults. Independent of chronic conditions or diseases, these complaints are associated with lower well-being. Therefore, they should be taken into account even in the absence of a significant disease. Pain, weakness, and fatigue are the most common and severe complaints reported among older adults. They are also more frequent in women and people aged 70 and over. The evaluation of physical complaints should be included in the routine geriatric assessments. More emphasis is needed for screening, evaluation, improvement, and relief of these complaints. It is also recommended to investigate trends in physical complaints in longitudinal and cohort studies.

## Data Availability

The dataset for this study can be obtained by making a reasonable request to the corresponding author.
